# Supplementary material for: Intertwined dipolar and multipolar order in the triangular-lattice magnet TmMgGaO4
Source: Nat Commun. 2019 Oct 8;10:4530. doi: 10.1038/s41467-019-12410-3 (PMC6783407; doi:10.1038/s41467-019-12410-3)
Supplement: Supplementary file 1 — Supplementary Information [file 41467_2019_12410_MOESM1_ESM.pdf]

Supplementary Information for

Intertwined dipolar and multipolar order in the triangular-lattice magnet $TmMgGaO_4$

Shen *et al.*

Supplementary Note 1: Sample synthesis and characterization.

High quality single-crystalline $TmMgGaO_4$ samples were synthesized using the floating-zone technique. The single crystal is transparent with shiny cleaved surfaces (Supplementary Fig. 1a). Sharp and clear diffraction spots can be seen in the X-ray Laue pattern (Supplementary Fig. 1b). In the single-crystal X-ray diffraction measurement, a series of reflections along the L direction can be indexed and the full-width at the half maximum (FWHM) of the rocking scan across **Q**=(0, 0, 18) is around 0.025 degrees (Supplementary Fig. 1c). These results indicate the high crystallization quality of the single crystals.

Supplementary Note 2: Calculations of the spin excitations in $TmMgGaO_4$.

In order to understand the observed spin excitation spectra in $TmMgGaO_4$ at low temperature, we calculate the spin wave dispersion by linear spin wave theory (LSW) using the SPINW program[1]. Here we present the calculated results of five representative scenarios.

First, we examine the possibilities of conventional dipolar ordering. The corresponding spin Hamiltonian is assumed as:

$$\mathcal{H} = \sum_{\langle ij \rangle}[J_1^{zz} S_i^z S_j^z + J_1^{\pm}(S_i^+ S_j^- + S_i^- S_j^+)] + \sum_{\langle\langle ij \rangle\rangle} J_2^{zz} S_i^z S_j^z \quad (1)$$

where $S^{\pm} = S^x \pm iS^y$ and J_1 and J_2 are nearest and next-nearest neighboured magnetic interactions, respectively, as illustrated in Supplementary Fig. 2a. The three-fold magnetic twins are included for all calculations.

We consider the isotropic Heisenberg model ($J_1^{zz} = 2J_1^{\pm} = 0.9$ meV, $J_2^{zz} = 0$ meV) and anisotropic XY model ($J_1^{zz} = J_2^{zz} = 0$ meV, $J_1^{\pm} = 0.4$ meV) that support the 120 degree Néel order and lead to strong magnetic Bragg peaks at K points (Supplementary Fig. 3a). We calculate both the total scattering function ($S_{xx} + S_{yy} + S_{zz}$) and the longitudinal component (S_{zz}) for both of the models (Supplementary Fig. 4a-d). Although the calculated spin waves catch the feature of Goldstone mode stemmed from K points, the branches around Γ points go as a minimum in the calculation instead of a maximum which is observed in the neutron experiments. Moreover, the branches around the M points are located at higher energies than the experimental data.

Another scenario is the Ising model with a stripe order that has been proposed in a recent research[2] (Supplementary Fig. 3b). The parameters are chosen to be $J_1^{zz} = 0.8$ meV and $J_2^{zz} = 0.076$ meV. The proposed stripe antiferromagnetic structure will lead to magnetic Bragg peaks at M points. We measured the elastic scattering at M points with the similar statistic with that at Γ point in the main text. As shown in Supplementary Fig. 5, no indication of peak-like feature can be distinguished. For the spin excitations, since all spins are aligned along the c direction, the S_{zz} sector vanishes for local spins and the transverse components are essentially dispersionless, clearly inconsistent with our data (Supplementary Fig. 4e).

In addition, we calculate the energy dependence of the spectral intensity at Γ and M points with various values of $J_1^{zz}/J_1^{\pm}$. It is shown that the simulated spin wave energy at the Γ point is either comparable or lower than that at the M point (Supplementary Fig. 6). This is inconsistent with our data where the spin wave energy at the Γ point exhibits a maximum with an energy

significantly higher than that at the M point (Fig. 3a). Thus, the anisotropic XXZ model with a dipolar order by no means can describe the spin wave dispersion in $TmMgGaO_4$.

We also calculate the scattering function based on the quasi-doublet scenario that is proposed in the main text (Supplementary Fig. 2b). We show that the longitudinal excitations (S_{zz} channel) of the intertwined multipolar order are in excellent agreement with our data (Fig. 2g-l, Fig. 3b).

Finally, we consider the intertwined multipolar order raised from a non-Kramers doublet system[3]. It is described by the Hamiltonian

$$\mathcal{H} = \sum_{\langle ij \rangle}[J_1^{zz}S_i^zS_j^z + J_1^{\pm}(S_i^+S_j^- + S_i^-S_j^+) + J_1^{\pm\pm}(\gamma_{ij}S_i^+S_j^+ + \gamma_{ij}^*S_i^-S_j^-)] + \sum_{\langle\langle ij \rangle\rangle} J_2^{zz}S_i^zS_j^z \quad (2)$$

where γ_{ij} are phase factors that depend on the directions of the bonds. In this scenario, the effective spin components S^x and S^y show hidden quadrupolar behavior while the out-of-plane component S^z shows dipolar behavior, similar to the quasi-doublet scenario that is proposed for $TmMgGaO_4$ in the main text. However, the observed large Landé g-factor (12.11) is inconsistent with a non-Kramers doublet state. Moreover, the heat capacity of the highly diluted $Tm_{0.04}Lu_{0.96}MgGaO_4$ shows a finite zero-temperature limit of C_m/T, which is consistent with the quasi-doublet state, but inconsistent with the non-Kramers doublet state[2].

Supplementary Note 3: Magnetic form factor.

For conventional dipolar moments, the magnetic form factor decreases with increasing **Q** while the multipoles may display an anisotropic and non-monotonic **Q**-dependence of the magnetic form factor, because multipoles have complex magnetization density[4]. In Supplementary Fig. 7, we present the constant energy slices of the simulated spin wave excitations considering a dipolar Tm^{3+} form factor. The simulated spin excitation signal in the second Brillouin zone is considerably weaker than that of the first zone, which is clearly inconsistent with the measured data in Fig. 2a-f. The constant energy cuts through the first and second Brillouin zones further prove that the spin excitation intensity does not follow the dipolar Tm^{3+} form factor (Supplementary Fig. 8).

Correspondence and requests for materials should be addressed to J.Z. (zhaoj@fudan.edu.cn) or G.C. (gchen_physics@fudan.edu.cn).

---

1 Toth, S. & Lake, B. Linear spin wave theory for single-Q incommensurate magnetic structures. *J. Phys.: Condens. Matter* **27,** 166002 (2015).

2 Li, Y., Bachus, S., Tokiwa, Y., Tsirlin, A. A. & Gegenwart, P. Absence of zero-point entropy in a triangular Ising antiferromagnet. Preprint at https://arxiv.org/abs/1804.00696 (2018).

[3] Liu, C., Li, Y.-D. & Chen, G. Selective measurements of intertwined multipolar orders: non-Kramers doublets on a triangular lattice.*Phys. Rev. B* **98,** 045119 (2018).

[4] Santini, P. *et al.* Multipolar interactions in f-electron systems: the paradigm of actinide dioxides. *Rev. Mod. Phys.* **81,** 807-863 (2009).

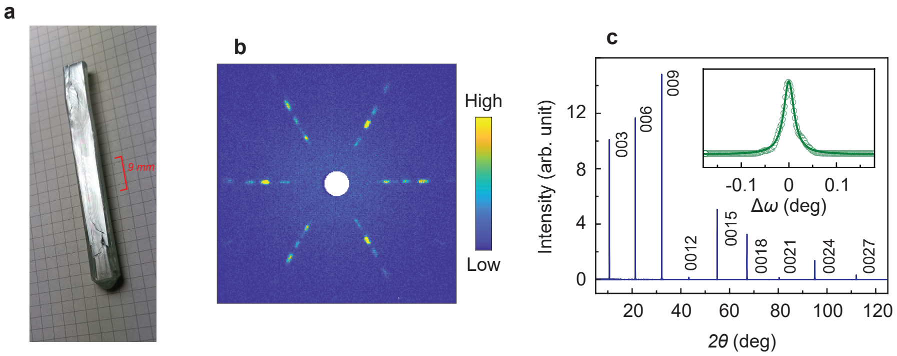

Supplementary Figure 1: **Photograph and X-ray diffraction patterns of $TmMgGaO_4$ single crystals. a,** A photograph of a representative $TmMgGaO_4$ single crystal. **b,** X-ray Laue pattern viewed from the *c* axis. The color bar indicates scattering intensity in arbitrary unit in linear scale. **c,** X-ray diffraction pattern from the cleaved surface of a $TmMgGaO_4$ single crystal. The inset shows the Lorentz fitting of the rocking curve of the (0, 0, 18) peak.

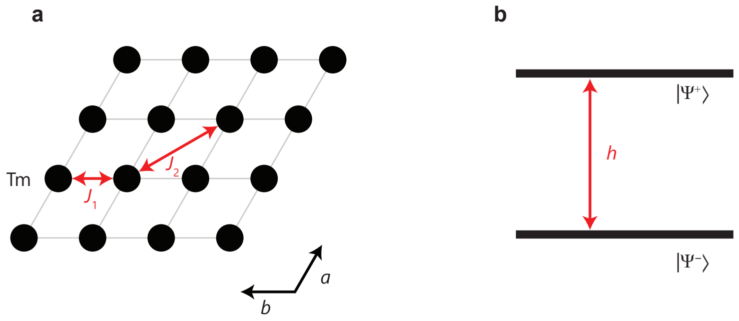

Supplementary Figure 2: **Sketch of the magnetic interactions and CEF splitting in $TmMgGaO_4$. a,** Illustration of the triangular lattice plane of Tm ions. The nearest (J_1) and next-nearest magnetic interactions (J_2) are marked by the red arrows. The grey solid lines indicate the unit cell. **b,** CEF splitting of the low-lying two singlet levels. h is the effective transverse field term that models the splitting between two singlet levels.

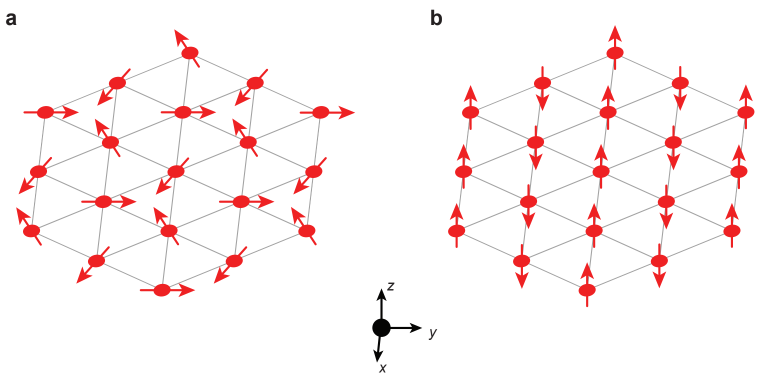

Supplementary Figure 3: **Mean-field spin configurations of the different states. a,** Illustration of the spin structure in 120 degree Néel order. All the spins lie in the *xy* plane. **b,** Magnetic structure of the stripe order. All the spins are aligned along *z* direction.

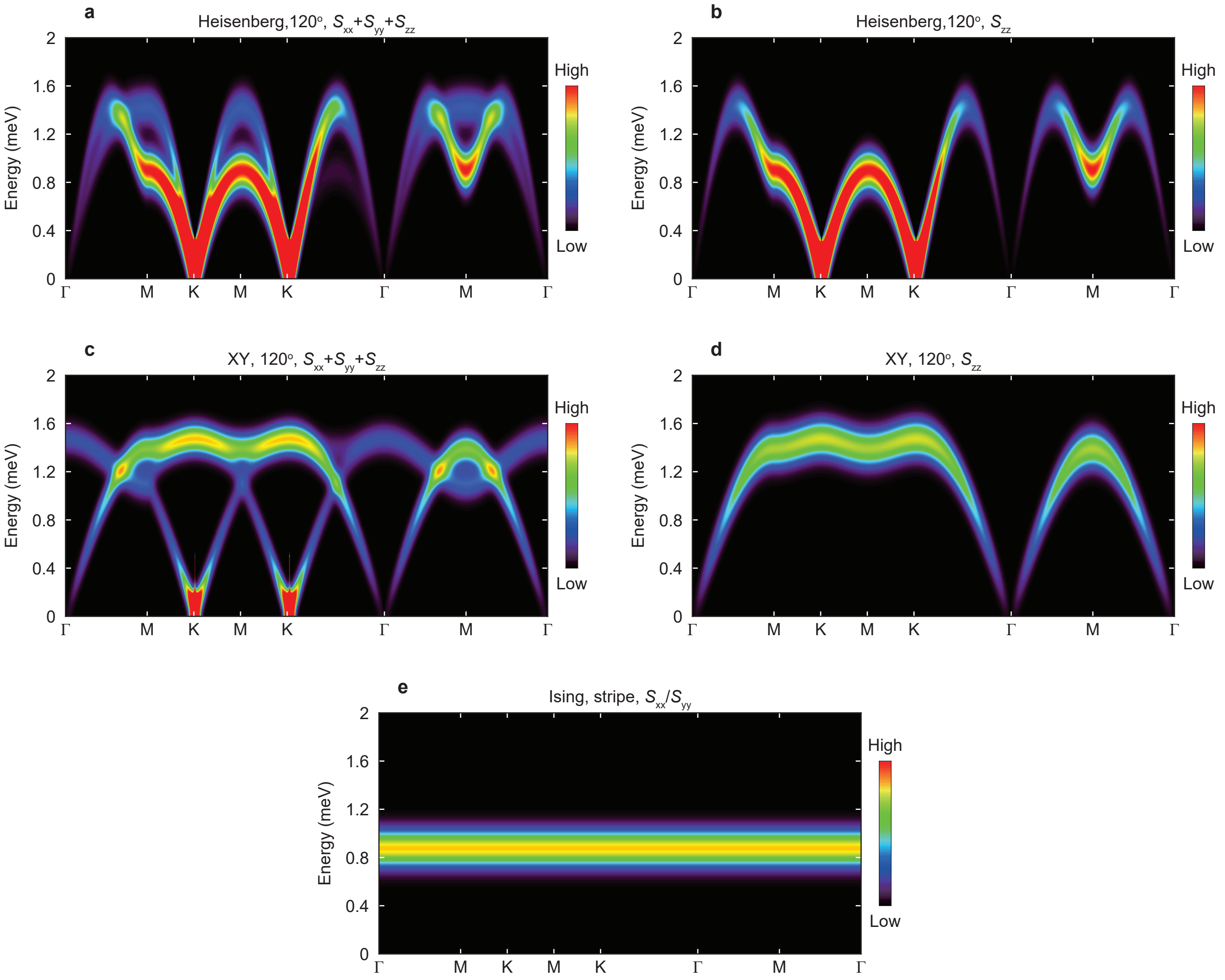

Supplementary Figure 4: **Calculated spin excitation dispersions with different models and scattering sectors. a, b,** Total and longitudinal scattering function for isotropic Heisenberg model. **c, d,** Total and longitudinal scattering function for XY model with the 120 degree magnetic structure. **e,** Transverse scattering sector for Ising model. The color bars indicate scattering intensity in arbitrary unit in linear scale.

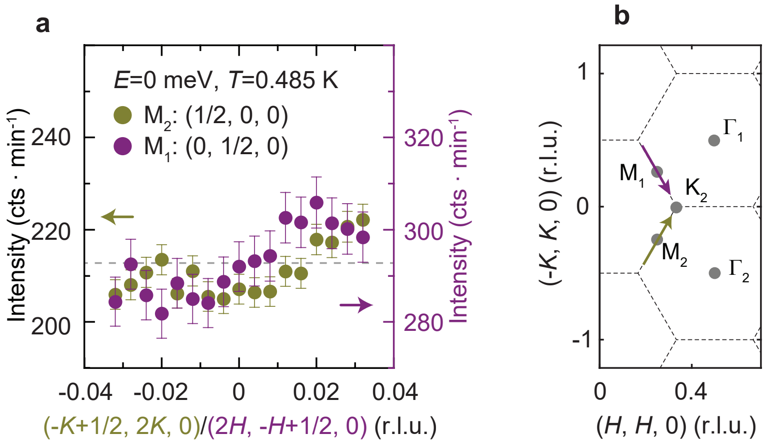

Supplementary Figure 5: **Absence of Bragg peaks at M points. a,** Constant energy cuts across M points at E=0 meV along the directions indicated by the yellow and purple arrows in **b**. Error bars, 1 s.d. **b**, Sketch of the reciprocal space.

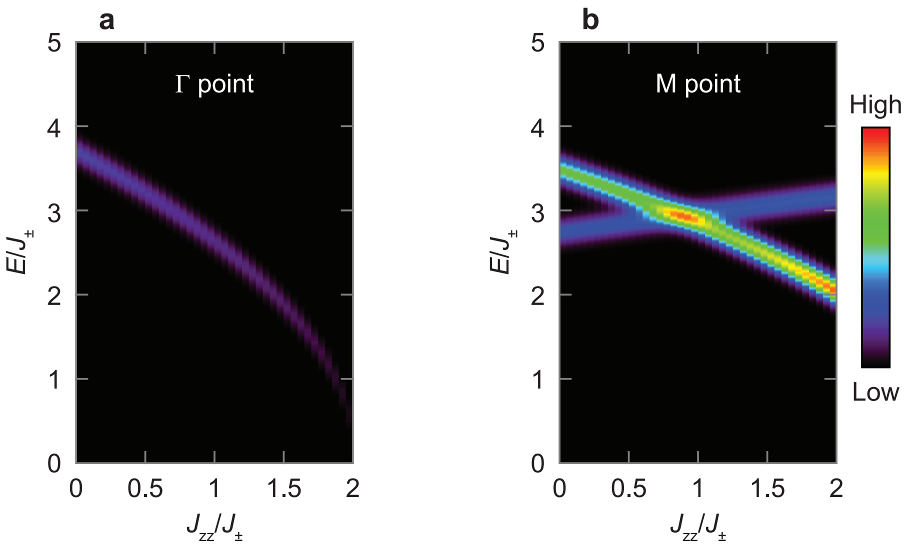

Supplementary Figure 6: **Energy dependence of the spectral intensity at high symmetry points with various magnetic interactions.** Γ point (**a**); M point (**b**). The color bar indicates scattering intensity in arbitrary unit in linear scale.

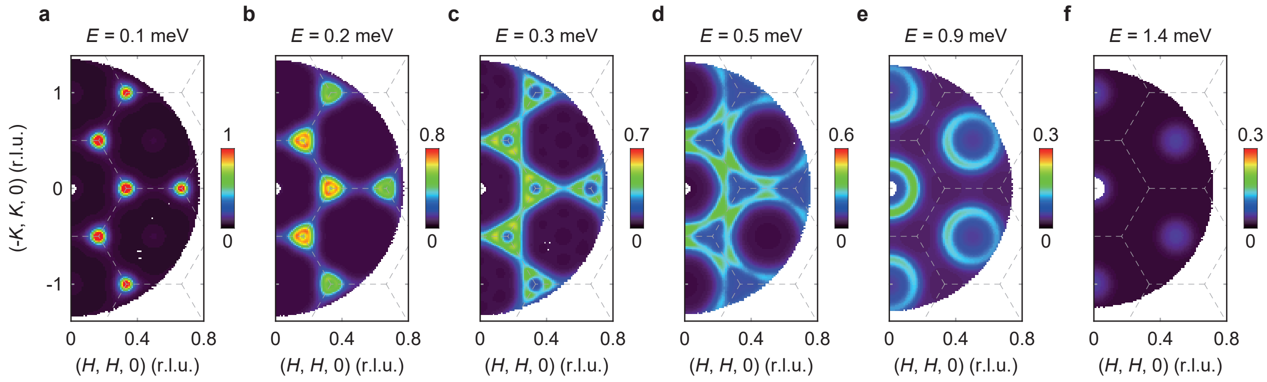

Supplementary Figure 7: **Calculated momentum dependence of spin excitations at various energies assuming a dipolar** $\mathbf{Tm^{3+}}$ **form factor.** E=0.1 meV (**a**); E=0.2 meV (**b**); E=0.3 meV (**c**); E=0.5 meV (**d**); E=0.9 meV (**e**); E=1.4 meV (**f**). The simulation is done with parameters specified in the main text. The color bars indicate scattering intensity in arbitrary unit in linear scale.

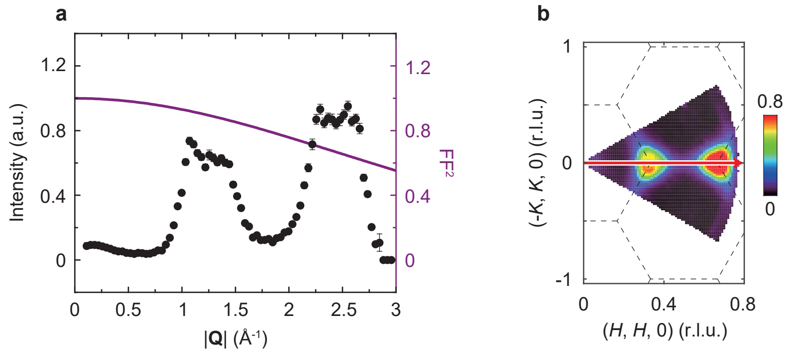

Supplementary Figure 8: ***Q*-dependence of the spin wave intensity. a,** Constant energy cuts at 0.05 K and 0.2 meV along the direction marked by the red arrow in **b**. The purple solid lines indicate the form factor of dipolar Tm^{3+} ions. **b,** Constant energy slice at 0.05 K and 0.2 meV. All the data are symmetrized into a 60 degree wedge of the reciprocal lattice. The color bar indicates scattering intensity in arbitrary unit in linear scale.
